# Hepatobiliary cystadenocarcinoma without mesenchymal stroma in a female patient: a case report

**DOI:** 10.1186/1471-230X-14-109

**Published:** 2014-06-16

**Authors:** Yang-Hong Dai, Yee Hui Yeo, Yao-Feng Li, Chung-Bao Hsieh, Yu-Lueng Shih

**Affiliations:** 1 Department of Internal Medicine, Tri-Service General Hospital, Taipei, Taiwan; 2 Department of Medicine, National Defense Medical Center, Taipei, Taiwan; 3 Department of Medical Education and Research, Taichung Veterans General Hospital, Taichung, Taiwan; 4 Department of Pathology, Tri-Service General Hospital, Taipei, Taiwan; 5 Division of General Surgery, Department of Surgery, Tri-Service General Hospital, National Defense Medical Center, Taipei, Taiwan; 6 Division of Gastroenterology, Department of Internal Medicine, Tri-Service General Hospital, National Defense Medical Center, Taipei, Taiwan

**Keywords:** Hepatobiliary cystadenocarcinoma, Biliary tumors, Hepatolithiasis, Cholestasis

## Abstract

**Background:**

Hepatobiliary cystadenocarcinoma is a rare epithelial malignant neoplasm of the liver or extrahepatic bile ducts. Early diagnosis of hepatobiliary cystadenocarcinoma is difficult because of its asymptomatic features and rarity. Moreover, the molecular pathogenesis of hepatobiliary cystadenocarcinoma remains unclear. Herein, we described a case of hepatobiliary cystadenocarcinoma in female with chronic hepatitis B and repeated hepatolithiasis.

**Case presentation:**

A 65-year-old woman with medical history of latent hepatitis B virus infection, repeated choledocholisthiasis, and cholecystitis was admitted due to a heterogeneous cystic mass (5.6 cm × 4 cm) shown on abdominal ultrasonography during regular physical checkup. The patient complained about irregular bowel movements with intermittent diarrhea for two months before presentation. Computed tomography (CT) disclosed a multiloculated cystic lesion in the left hepatic lobe with the presence of intraductal stones and dilatation of intrahepatic ducts. Histological results obtained from left lobectomy specimens showed hepatobiliary cystadenocarcinoma without accompanied mesenchymal stroma.

**Conclusion:**

Notably, hepatobiliary cystadenocarcinoma without mesenchymal stroma seldom occurs in women and is usually associated with poor prognosis. We present the rare findings in this patient and suggest that chronic inflammatory insults in the intrahepatic bile ducts might shed light on the cystadenocarcinogenesis.

## Background

Hepatobiliary cystadenocarcinoma is a rare cystic neoplasm that predominantly occurs in the middle-aged women. Its early detection is challenging because of varied clinical features [1]. The tumor may originate from pre-existing cystadenoma or from other benign cystic lesions. However, it can also develop “de novo” from the biliary epithelium or peribiliary glands [2]. The two subgroups, cystadenocarcinoma with mesenchymal stroma and without stroma may yield different clinical outcomes [3]. The presence of mesenchymal stroma that resembles ovarian stroma (OS) suggests the possible etiology during embryonic development and its female preponderance [4]. In the context of OS, cystadenoma is usually the prerequisite for cystadenocarcinoma. On the contrary, the mechanisms of cystadenocarcinogenesis for subtype without OS remains unclear. Chronic inflammation stimulated by hepatolithiasis may play a significant role in driving the malignant transformation of cholangiocarcinoma [5] and intraductal papillary neoplasia of the liver [6]. We suspect that this may also associate with the carcinogenesis of hepatobiliary cystadenocarcinoma. We report here on a case of a hepatobiliary cystadenocarcinoma without mesenchymal stroma and discuss the involvement of chronic inflammatory assailants caused by hepatholithiasis, chronic HBV infection, and surgical intervention.

## Case presentation

A 65-year-old woman was admitted to our hospital for surgical intervention due to one liver tumor identified on the abdominal ultrasonography during regular follow-up for chronic hepatitis B. Before presentation, the patient reported to have irregular bowel movement with loose stool passage and weight gain for about two months. She experienced no abdominal pain, fullness or vomiting. When tracing her past history, the patient had undergone cholecystectomy for cholecystitis and received two times of laparotomy in regard to repeated choledocholisthiasis caused by hepatolithiasis about 30 and 20 years ago, respectively. Besides, four of the patient’s seven siblings were diagnosed with hepatitis B-related hepatocellular carcinoma, which had contributed to their death and severe morbidity.

**Figure 1:**
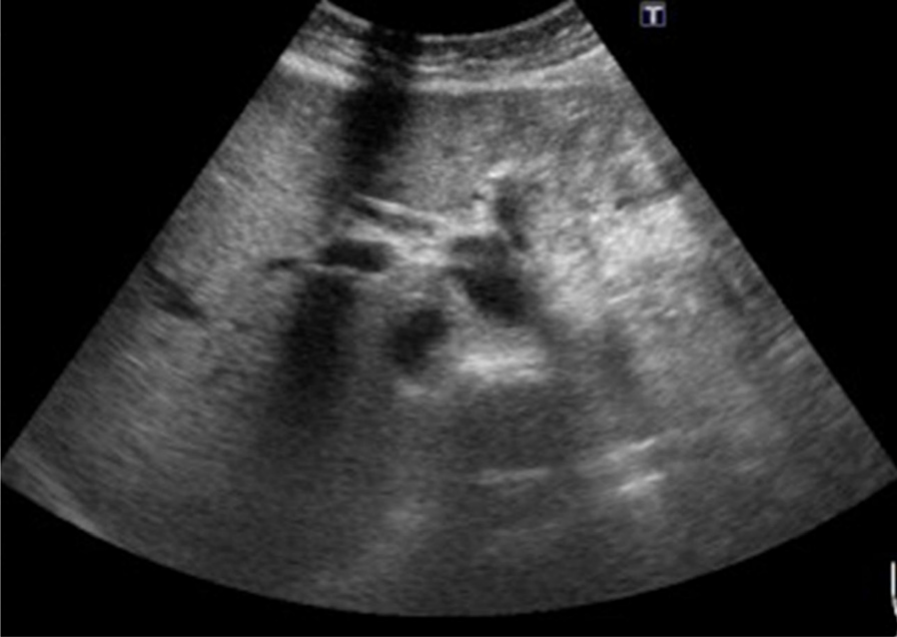
**Abdominal ultrasonography.** Abdominal ultrasonography (US) showed a multiloculated cystic mass over the lateral segment of left hepatic lobe.

**Figure 2:**
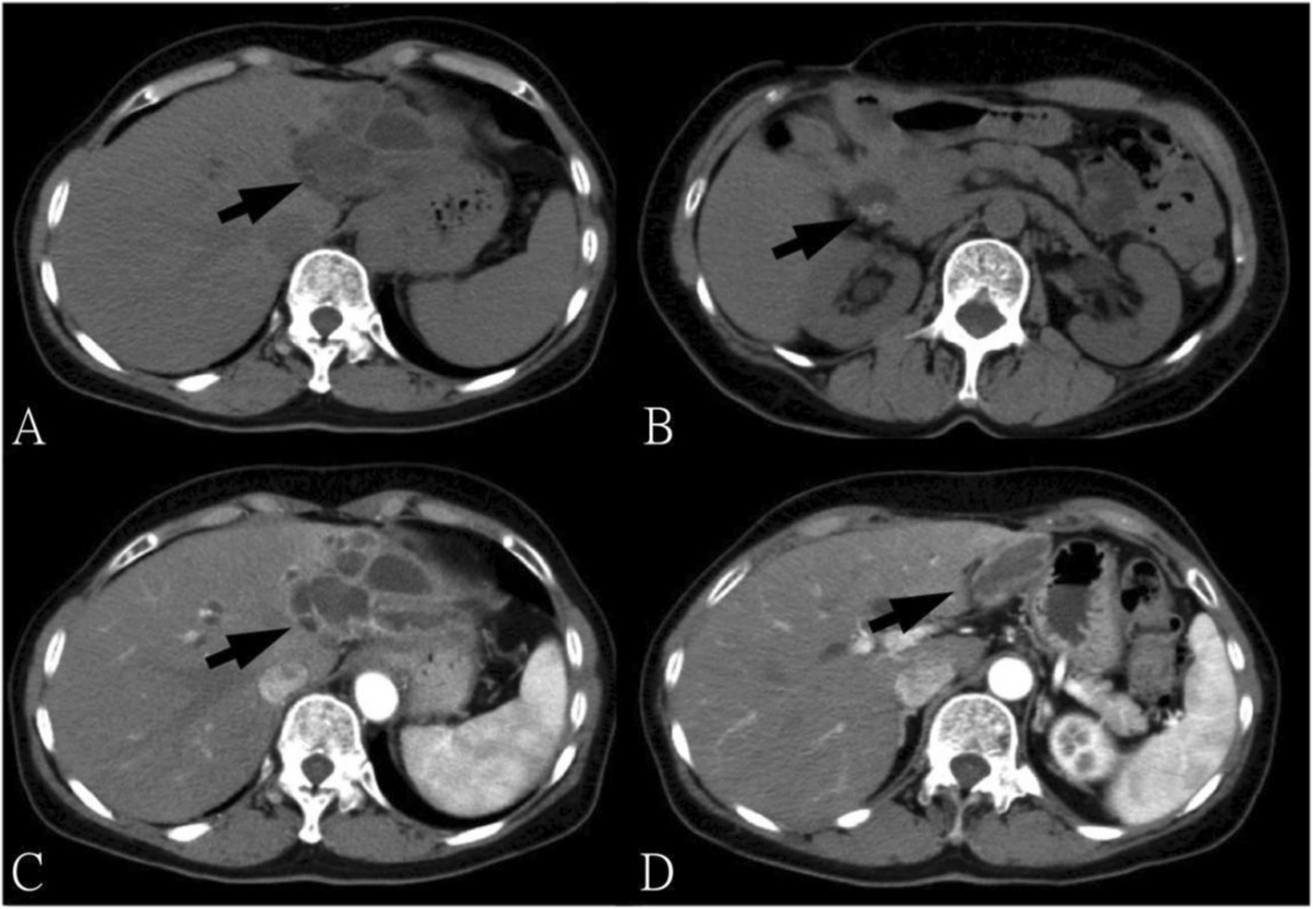
**Computed tomography of the liver and bile duct. (A)** Computed tomography (CT) disclosed multiloculated cystic lesion with internal septa (arrow). **(B)** Gallbladder stone was identified (arrow). **(C)** After the administration of contrast medium, there was enhancement delineating the contour of the cystic tumor (arrow). **(D)** The cystic lesion extended to the lateral division of the left hepatic lobe (arrow).

**Figure 3:**
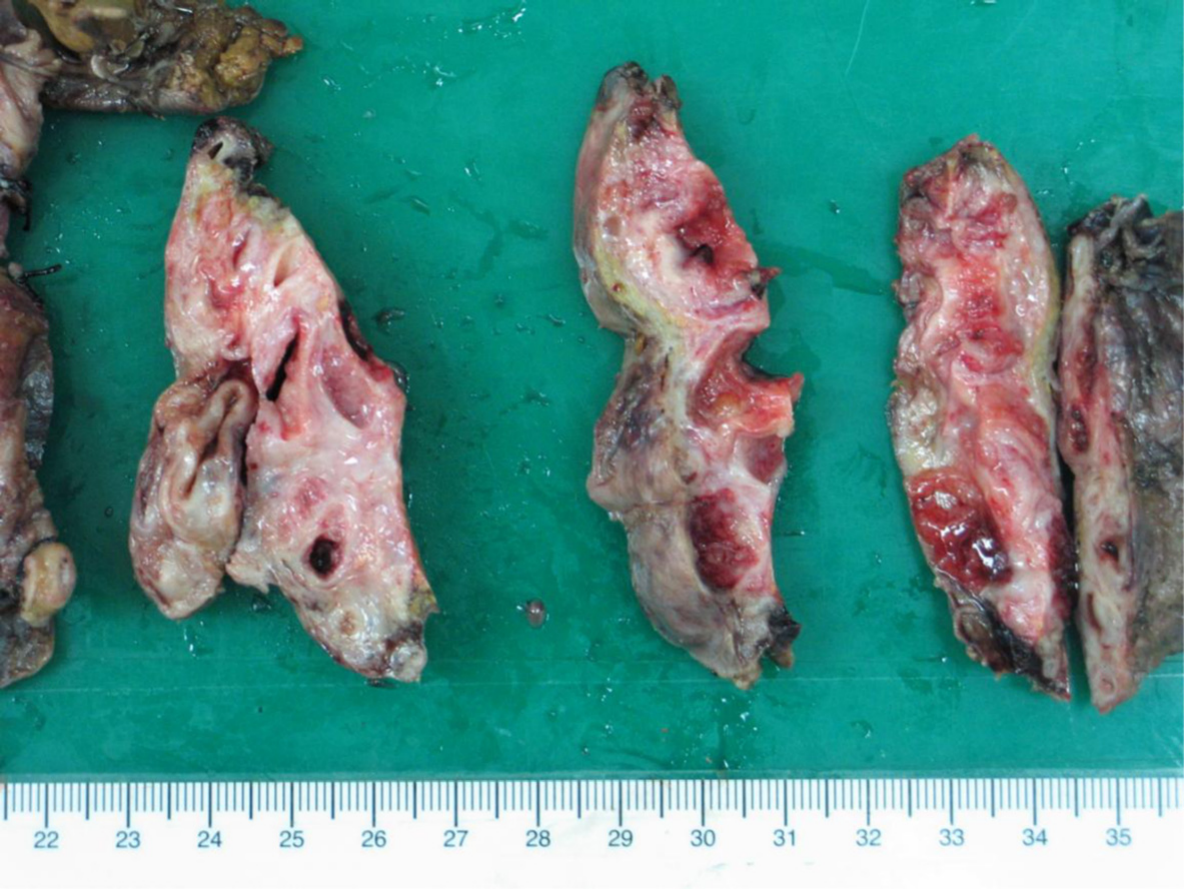
**Gross section of the cystic tumor.** The resected mass showed solitary, multi-cystic and ill-defined appearance with about 0.1 cm of distance beneath the liver capsule.

**Figure 4:**
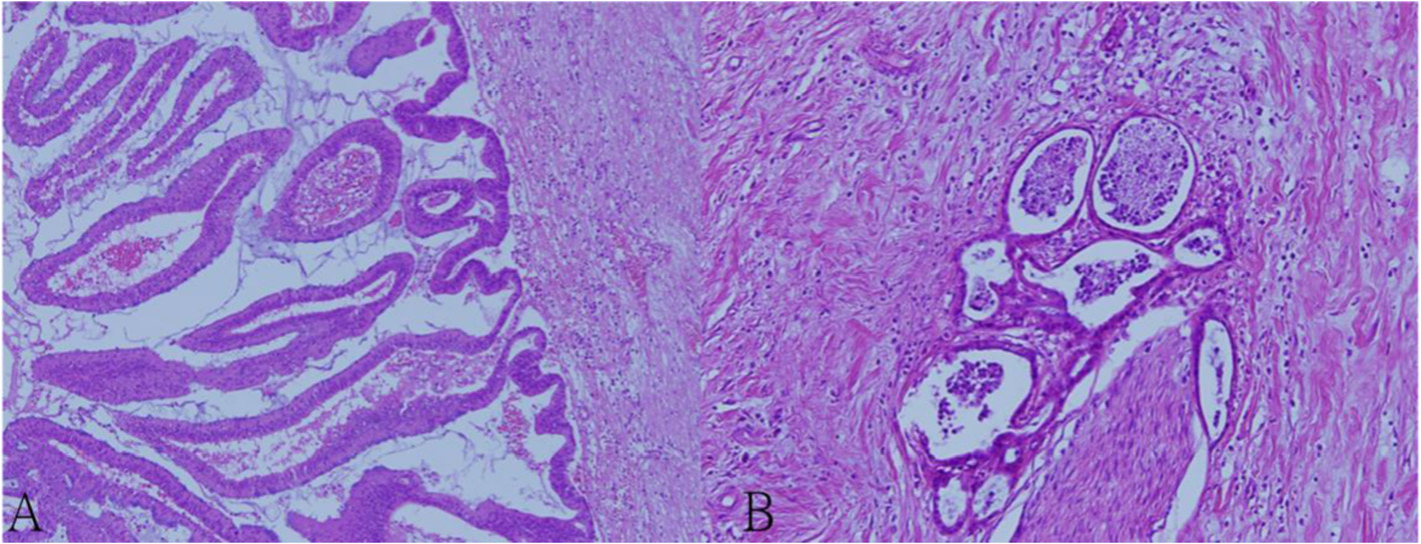
**Pathological findings from the resected lesion. (A)** Photograph of the pathologic specimen shows well-differentiated biliary cystadenocarcinoma characterized by columnar cells arranged in papillary structures (original magnification × 100). Note that there is no ovarian stroma present. **(B)** Perineural invasion of the tumor cells is observed in some parts of the tumor (hematoxylin & eosin staining, original magnification × 200).

The physical examination was unremarkable. There was no abdominal tenderness, rebounding tenderness and abnormal bowel sounds. Murphy’s sign was also negative. Laboratory studies on admission yield normal blood biochemistry and mild thrombocytopenia (127000 per cubic millimeter). The liver function test showed normal results (AST = 19 U/L, ALT = 16 U/L). No prolonged prothrombin times or partial-thromboplastin times was found. Direct and total bilirubin were not elevated. Enzymes such as gamma-glutamyl transferase or alkaline phosphatase were within normal ranges. The urinalysis was normal, without dark or tea-colored urine. The levels of tumor markers such as AFP, CEA, CA125 and CA19-9 were all within normal limits.Ultrasonography (Figure 1) disclosed a heterogeneous cystic lesion about 7.6 cm × 5.5 cm over the lateral segment of left hepatic lobe with dilatation of intrahepatic ducts. Computed tomography (CT) of abdomen demonstrated multiloculated cystic tumor with internal septa in the left lobe of the liver (Figure 2). In addition, non-calcified stones were found in dilated intrahepatic bile duct and common bile duct. Under the impression of cholangiocarcinoma, left lobectomy of liver and choledocholithotomy were performed. The cystic tumor was smoothly removed with clear margin. Grossly, the resected tumor demonstrated multi-cystic and ill-defined appearance beneath the liver capsule (Figure 3). All sections were embedded and examined. Microscopically, a cyst lined by proliferating epithelia composed of papillary proliferation of dysplastic cells with mucin hypersecretion was identified (Figure 4A). It was supported by fibromuscular structure where ovary-like stroma was not seen. Furthermore, malignant picture with deregulated growth pattern and perineural invasion was demonstrated in some regions (Figure 4B). The cystic tumor was finally diagnosed as a hepatobiliary cystadenocarcinoma without mesenchymal stroma (pT1N0M0).

After total resection of the tumor, the patient was hospitalized for several days. She had no fever, no distention and other morbidities before discharge. Her incision had healed well 3 weeks after discharge. She showed no adverse event related to hepatobiliary disease or the surgery. The follow up MRI three years after operation confirmed no recurrence of malignancy.

## Discussion

Hepatobiliary cystadenocarcinoma is a rare cystic neoplasm that usually presents as a multilocular cystic mass with internal septations, nodules, and irregular, thickened cyst wall [1,7,8]. It may occur at any age with females affected more than males. Though the exact etiology remains uncertain, it has been proposed that the majority of these tumors come from a pre-existing cystadenoma or benign bile duct cysts [2,9]. In addition, some studies suggested that the cystic adenocarcinomas are the cystic variants of cholangiocarcinoma with shared biliary phenotypes [1]. This makes the clinicopathological features more varied than its counterpart, cystadenomas alone. The cystic tumors in females that frequently present ovarian-like stroma (OS) are not observed in male cases [1]. It may be due to the proximity between liver and gonads during embryonic development and tissue migration [4], which can explain why cystadenoma and cystadenocarcinoma with OS exclusively occurs in women. In addition, the expression of estrogen and progesterone receptors in the stromal cells also demonstrates the female predominance in the disease series [10]. CA 19–9, a marker that is elevated in gastrointestinal cancer and bile duct diseases, has been previously reported to be elevated in hepatobiliary cystadenocarcinoma with OS [11] but within normal range in its counterpart without OS [12]. This is manifested in the present case, which further implies the difference between these two subtypes. Clinically, the presence of mesenchymal stroma that resembles OS indicates a relatively indolent course and a better prognosis [3]. However, the malignant potential of this subtype engender the necessity for total resection. On the contrary, cystadenocarcinomas without mesenchymal stroma occur in both genders and present worse outcomes. Two larger studies revealed the survival rate of cystadenocarcinomas without OS is not more than 50% [1,2]. In our case, however, the patient who presents hepatobiliary cystadenocarcinoma without OS remains in healthy status three years after surgery.

There is currently little evidence describing the genesis of cystadenocarcinoma without OS. More clinical and pathological studies are required to determine whether it is generated directly from biliary epithelium or from other pre-malignant structures. One possible mechanism of the initial cystic formation comes from the hyperplasia of peribiliary glands which are located along the walls of extrahepatic ducts, the neck of the gallbladder and the larger intrahepatic bile ducts [13,14]. These glands are proposed to serve as source of stem cells for biliary epithelia regeneration under normal condition and in response to injury [14]. Therefore, it is reasonable to deduce that pathological insults to these glands may instigate a variety of disorders including cancer. In support of this, hepatobiliary diseases such as cirrhosis, cholangitis and hepatolithiasis are correlated with the hyperplasia of peribiliary glands [1,5,15]. In the present case, multiple insults including surgeries [16] due to hepatolithiasis, chronic HBV infection, and IHD stones may synergistically irritate peribiliary glands thereby causing progressively pathological changes and poses risk for malignant changes. In recent years, an entity named intraductal papillary mucinous neoplasms (IPMN) has been recognized as a unique neoplasm arising from intrahepatic biliary tree [17]. It is characterized by solitary and diffuse growth of biliary epithelia into the bile ducts with a papillary pattern. Hepatholithiasis has been suggested to initiate the genesis of IPMN [18]. Chen et al. [6] reported that chronic inflammation in the context of hepatolithiasis causes papillary growth of the biliary epithelia with subsequent metaplasia and invasive carcinoma. Intrahepatic duct stone and intracystic stone formation has been reported to involve in biliary cystadenoma or cystadenocarcinoma [19,20]. Importantly, Tseng reported a case of biliary cystadenocarcinoma featured by hepatholithiasis and bilateral IHD and CBD stones, which might also contribute to a microenvironment of chronic inflammation [21]. Collectively, these findings converge on the conclusion that chronic stimulation of bile materials and calculi may exert negative influence and thereby cause a variety of hepatobiliary disorders.

In addition to the presence of calculi in hepatolithiasis, cholestasis is the common phenomenon of cirrhosis, cholangitis and bile duct cysts. To prove the effect of cholestasis on the biliary epithelium, Yang and colleagues [22] induced cholestasis in mouse with chemicals such as carbon tetrachloride or dimethylnitrosamine (DMN) and bile duct ligation (BDL). These approaches accelerated progression of cholangiocarcinoma through down-regulation of miR-34a and let-7a as well as up-regulation of Lin-28B. Of particular note, the up-regulation of Lin-28B is associated with the development of cystic hyperplasia of intrahepatic bile ducts. Moreover, atypical cystic hyperplasia was induced by cholestasis in the progression of cholangiocarcinoma. Taken together, the correlation between the cystic variant of cholangiocarcinoma and cystadenocarcinoma seems to lie in the cholestasis-mediated cystic formation. The authors suggest that cystadenocarcinoma could be caused by cholestasis based on the similar mechanisms.

To avoid development of biliary tract malignancies due to surgical intervention, anatomic hepatectomy [23] and laparoscopic right hemihepatectomy [24] could be performed to treat hepatolithiasis.

## Conclusion

In conclusion, we strongly suspect that hepatolithiasis and other inflammatory changes contribute to the formation of the patient’s hepatobiliary cystadenocarcinoma, probably through hyperplasia and neoplasia of peribiliary glands and biliary epithelia. It is a possible etiology other than benign cystic lesion or ectopic ovarian tissue during embryonic development, and the absence of ovarian stroma also lowers the possibility of tumorigenesis from pre-existing cystadenoma. Furthermore, since the absence of OS has poorer clinical outcomes, early detection and complete resection of this tumor are the key factors for achieving cure. Regular ultrasonography checkup is warranted for patient with evidence of hepatholithiasis and associated dilatation of intrahepatic ducts for early diagnosis of this malignant disease.

## Consent

Written informed consent was obtained from the patient for publication of this case report and any accompanying images. A copy of the written consent is available for review by the Editor of this journal.

## Competing interests

The authors declared that they have no competing interests.

## Authors’ contributions

YHD and YHY co-wrote the paper; CBH performed surgical operation; YFL performed pathological analysis; all authors cared for the patient and provided advice on the clinical aspects of the case report. All authors read and approved the manuscript.

## Pre-publication history

The pre-publication history for this paper can be accessed here:

http://www.biomedcentral.com/1471-230X/14/109/prepub
